# Surveillance for Ebola Virus in Wildlife, Thailand

**DOI:** 10.3201/eid2112.150860

**Published:** 2015-12

**Authors:** Supaporn Wacharapluesadee, Kevin J. Olival, Budsabong Kanchanasaka, Prateep Duengkae, Supakarn Kaewchot, Phimchanok Srongmongkol, Gittiyaporn Ieamsaard, Patarapol Maneeorn, Nuntaporn Sittidetboripat, Thongchai Kaewpom, Sininat Petcharat, Sangchai Yingsakmongkon, Pierre E. Rollin, Jonathan S. Towner, Thiravat Hemachudha

**Affiliations:** King Chulalongkorn Memorial Hospital and Faculty of Medicine, Chulalongkorn University, Bangkok, Thailand (S. Wacharaplluesadee, N. Sittidetboripa, T. Kaewpom, S. Petcharat, S. Yingsakmongkon, T. Hemachudha);; EcoHealth Alliance, New York, New York, USA (K.J. Olival);; Department of National Parks, Bangkok (B. Kanchanasaka, S. Kaewchot, P. Srongmongkol, G. Ieamsaard, P. Maneeorn);; Kasetsart University Faculty of Forestry, Bangkok (P. Duengkae);; Centers for Disease Control and Prevention, Atlanta, Georgia, USA (P.E. Rollin, J.S. Towner)

**Keywords:** Ebola virus, bats, macaques, Thailand, surveillance, viruses, wildlife, zoonoses

**To the Editor:** Active surveillance for zoonotic pathogens in wildlife is particularly critical when the pathogen has the potential to cause a large-scale outbreak. The recent outbreak of Ebola virus (EBOV) disease in West Africa in 2014 was initiated by a single spillover event, followed by human-to-human transmission (*1*). Projection of filovirus ecologic niches suggests possible areas of distribution in Southeast Asia (*2*). Reston virus was discovered in macaques exported from the Philippines to the United States in 1989 and in sick domestic pigs in the Philippines in 2008 (with asymptomatic infection in humans) (*3*). Dead insectivorous bats in Europe were found to be infected by a filovirus, similar to other members of the genus *Ebolavirus* (*4*).

Although EBOV has historically been viewed as a virus from Africa, recent studies found that bat populations in Bangladesh and China contain antibodies against EBOV and Reston virus recombinant proteins, which suggests that EBOVs are widely distributed throughout Asia (*5*,*6*). Thus, an outbreak in Asian countries free of EBOV diseases may not only be caused by importation of infected humans and/or wildlife from Africa but may arise from in-country filovirus–infected wildlife. Serologic and molecular evidence for filoviruses suggests that members of the order Chiroptera (bats) may be their natural reservoir (*7*).

As part of a proactive biosurveillance program, we conducted a cross-sectional study for EBOV infection in bats and macaques in Thailand. We screened 500 *Pteropus lylei* bats collected from 10 roosting sites during March–June 2014 (Technical Appendix Figure) for antibodies against EBOV antigen by using an ELISA validated by the Centers for Disease Control and Prevention (Atlanta, GA, USA) (*8*).

Bats and macaques were captured with permission from the Department of National Parks, Wildlife and Plant Conservation. The Institutional Animal Care and Use Committee at the University of California, Davis (protocol #16048) approved the capture and sample collection protocols.

To further screen a wide range of wildlife species in Thailand for active EBOV infection, we sampled and tested 699 healthy bats, representing 26 species, and 50 long-tailed macaques (*Macaca fascicularis*). Additional bat species were randomly captured (≥50/site) in 6 provinces in Thailand during 2011–2013 and identified by morphologic traits. Macaques were captured and sampled in March 2013 from 1 site at Khao Chakan, Sa Kaeo Province, and released at the same site. Blood, saliva, urine, and feces were collected from anesthetized macaques or nonanesthetized bats. All animals were released after sample collection. Details on species screened, sample sizes, and trapping localities are provided in the Table. 

**Table:** Overview of bats and macaques tested by Ebola virus IgG ELISA or PCR for filoviruses, Thailand, 2011–2014

Species	Host family	No. tested (no. positive)	Test method*	Specimen type†	Location‡
Chiroptera					
* Pteropus lylei*	Pteropodidae	500 (0)	ELISA	Serum	a
* Cynopterus brachyotis*	Pteropodidae	10 (0)	PCR	Pooled	b
* C. sphinx*	Pteropodidae	4 (0)	PCR	Pooled	b
* Eonycteris spelaea*	Pteropodidae	12 (0)	PCR	Pooled	b
* Macroglossus sobrinus*	Pteropodidae	2 (0)	PCR	Pooled	b
* Megaerops niphanae*	Pteropodidae	1 (0)	PCR	Pooled	b
* Rousettus amplexicaudatus*	Pteropodidae	3 (0)	PCR	Pooled	b
* Hipposideros armiger*	Hipposideridae	113 (0)	PCR	Pooled	b
* H. cineraceus*	Hipposideridae	4 (0)	PCR	Pooled	b
* H. larvatus*	Hipposideridae	33 (0)	PCR	Pooled	b, c
* H. lekaguli*	Hipposideridae	158 (0)	PCR	Pooled	b
* Megaderma lyra*	Megadermatidae	1 (0)	PCR	Pooled	b
* Miniopterus magnate*	Vespertilionidae	132 (0)	PCR	Pooled	b, c
* M. pusillus*	Vespertilionidae	1 (0)	PCR	Pooled	b
* M. schreibersii*	Vespertilionidae	22 (0)	PCR	Pooled	b
* Myotis horsfieldi*	Vespertilionidae	6 (0)	PCR	Pooled	b
* M. muricola*	Vespertilionidae	1 (0)	PCR	Pooled	b
* Rhinolophus shameli*	Rhinolophidae	44 (0)	PCR	Pooled	b
* R. coelophyllus*	Rhinolophidae	7 (0)	PCR	Pooled	c
* R. luctus*	Rhinolophidae	1 (0)	PCR	Pooled	b
* R. malayanus*	Rhinolophidae	4 (0)	PCR	Pooled	c
* R. microglobosus*	Rhinolophidae	1 (0)	PCR	Pooled	b
* R. pusillus*	Rhinolophidae	1 (0)	PCR	Pooled	b
* Scotophilus kuhlii*	Vespertilionidae	1 (0)	PCR	Pooled	b
* Taphozous longimanus*	Emballonuridae	27 (0)	PCR	Pooled	b
* T. melanopogon*	Emballonuridae	110 (0)	PCR	Pooled	b
Total		699 (0)			
Primate					
* Macaca fascicularis*	Cercopithecidae	50 (0)	PCR	Pooled	d

*ELISA for IgG against Ebola virus. †Nucleic acid extraction from Pooled saliva, serum, and urine. ‡a, Central Thailand; b, Eastern Thailand; c, Chaing Mai Province; d, Kao Chakan, Sa Kaeo Province.

All nonblood specimens were collected in nucleic acid extraction buffer (lysis buffer) and transported on ice to the World Health Organization Collaborating Centre for Research and Training on Viral Zoonoses laboratory (Bangkok, Thailand) for storage and testing. Three types of specimen (saliva, urine, and serum) were collected from individual animals and pooled.

Nucleic acid was then extracted with NucliSENS easyMAG (bioMérieux, Boxtel, the Netherlands) and analyzed by reverse transcription PCR (RT-PCR). A consensus RT-PCR was used to screen for all known species of Ebola virus and Marburg virus, including EBOV (*9*). In total, 5 RT-PCRs were performed on each specimen, a regimen that included 4 sets of primers specific to known filoviruses and 1 degenerate primer set to detect novel viruses in this family. The sensitivity of RT-PCR on synthetic standard was 50–500 copies/reaction (*9*). We ran 3,745 PCRs, covering a range of assays, to increase detection sensitivity. All specimens examined were negative for filoviruses by EBOV ELISA and PCR (Table). For *P. lylei* ELISA screening, optical density values for all 500 bats ranged from 0.000 to 0.095, well below the potential positive cutoff value of 0.2. 

Assuming a population size of ≈5,000 bats/roost and a sample size of 50 bats/site, we have 95% confidence that if >6% of the population had antibodies against EBOV antigen, we would have detected it. If we assume that all 500 animals are part of 1 large panmictic population, and we have 95% confidence that if EBOV were circulating in >0.5% of the population, we would have detected it. Therefore, although we cannot rule out infection of this species with 100% confidence, *P. lylei* bats, the most abundant species of large pteropid bats in Thailand, are highly unlikely to be reservoirs for EBOV. 

Our sample sizes for PCR screening of other bat species in this study were much smaller, and we had no supported serologic data, but these negative results could add to the knowledge of filovirus infection in nontissue specimens from healthy bats. Previous studies have detected Ebola virus–like filovirus RNA in lung tissue of healthy *Rousettus leschenaultia* bats in China (*10*) and from organs and throat and rectal swab specimens from a die-off of *Miniopterus schreibersii* bats in Spain (4). In our study, which included 22 *M. schreibersii* and 132 *M. magnate* bats, none of the bats tested positive for filoviruses.One limitation of the cross-sectional sampling strategy used here, however, is that PCR-negative findings do not necessarily mean that the bats were not infected in the past. Although we found no evidence of filovirus infection in wildlife species tested in Thailand, we believe that continuing targeted surveillance in wildlife should enable early detection and preparedness to preempt emerging zoonoses.

**Technical Appendix.** Map showing 20 *Pteropus lylei* bat roosting sites (gray circles, update 2015) in Thailand from 10 years of population surveys by the Department of National Parks, Wildlife and Plant Conservation and Kasetsart University, Thailand. These bats form large, colonial aggregations of individual animals, which often roost near human dwellings and primarily in the central region of the country. The map shows that populations of this species are concentrated in Central Thailand. Ten sampling sites (black star) included in the current study, March–June 2014, were selected on the basis of the size of the bat population, >2,000 bats/colony (50 individual bats sampled/locality). Abbreviations indicate provinces where *P. lylei* bats were found: AT, Ang Thong; AY, Phra Nakhon Si Ayutthaya; BK, Bangkok; CH, Chonburi; CHS, Chachoengsao; NY, Nakhon Nayok; PBR, Prachinburi; SAK, Srakaeo; SB, Saraburi; SH, Singburi; SMR, Samut Sakhon; SP, Suphan Buri.
