# Supplementary material for: Surveillance for Ebola Virus in Wildlife, Thailand
Source: Emerg Infect Dis. 2015 Dec;21(12):2271–3. doi: 10.3201/eid2112.150860 (PMC4672430; doi:10.3201/eid2112.150860)
Supplement: Supplementary file 1 — Technical Appendix. Map showing 20 Pteropus lylei bat roosting sites (gray circles, update 2015) in Thailand from 10 years of population surveys by the Department of National Parks, Wildlife and Plant Conservation and Kasetsart University, Thailand. These bats form large, colonial aggregations of individual animals, which often roost near human dwellings and primarily in the central region of the country. The map shows that populations of this species are concentrated in Central Thailand. Ten sampling sites (black star) included in the current study, March–June 2014, were selected on the basis of the size of the bat population, >2,000 bats/colony (50 individual bats sampled/locality). Abbreviations indicate provinces where P. lylei bats were found: AT, Ang Thong; AY, Phra Nakhon Si Ayutthaya; BK, Bangkok; CH, Chonburi; CHS, Chachoengsao; NY, Nakhon Nayok; PBR, Prachinburi; SAK, Srakaeo; SB, Saraburi; SH, Singburi; SMR, Samut Sakhon; SP, Suphan Buri. [file 15-0860-Techapp-s1.pdf]

Article DOI: http://dx.doi.org/10.3201/eid2112.150860

Surveillance for Ebola Virus in Wildlife, Thailand

Technical Appendix

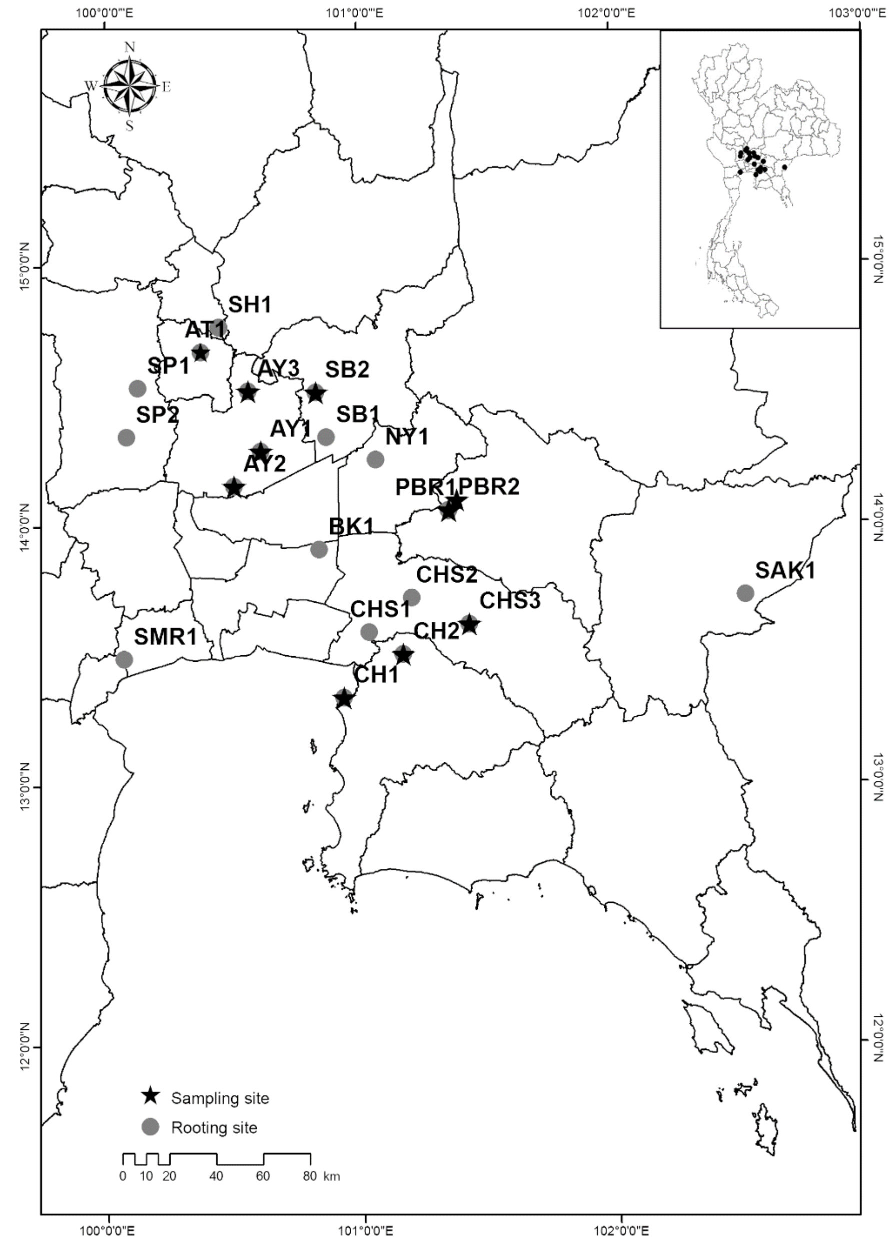

Technical Appendix Figure. Map showing 20 *Pteropus lylei* bat roosting sites (gray circles, update 2015) in Thailand from 10 years of population surveys by the Department of National Parks, Wildlife and Plant Conservation and Kasetsart University, Thailand. These bats form large, colonial aggregations of individual animals, which often roost near human dwellings and primarily in the central region of the country. The map

shows that populations of this species are concentrated in Central Thailand. Ten sampling sites (black star) included in the current study, March–June 2014, were selected on the basis of the size of the bat population, >2,000 bats/colony (50 individual bats sampled/locality). Abbreviations indicate provinces where *P. lylei* bats were found: AT, Ang Thong; AY, Phra Nakhon Si Ayutthaya; BK, Bangkok; CH, Chonburi; CHS, Chachoengsao; NY, Nakhon Nayok; PBR, Prachinburi; SAK, Srakaeo; SB, Saraburi; SH, Singburi; SMR, Samut Sakhon; SP, Suphan Buri.
